# Using Literature Based Discovery to Gain Insights Into the Metabolomic Processes of Cardiac Arrest

**DOI:** 10.3389/frma.2021.644728

**Published:** 2021-06-25

**Authors:** Sam Henry, D. Shanaka Wijesinghe, Aidan Myers, Bridget T. McInnes

**Affiliations:** ^1^ Department of Physics, Computer Science and Engineering, Christopher Newport University, Newport News, VA, United States; ^2^ Department of Pharmacotherapy and Outcomes Science, Virginia Commonwealth University, Richmond, VA, United States; ^3^ Department of Computer Science, Virginia Commonwealth University, Richmond, VA, United States

**Keywords:** literature based discovery, metabolomics, knowledge discovery, text mining, natural language processing, lipidomics, new discovery proposal

## Abstract

In this paper, we describe how we applied LBD techniques to discover lecithin cholesterol acyltransferase (LCAT) as a druggable target for cardiac arrest. We fully describe our process which includes the use of high-throughput metabolomic analysis to identify metabolites significantly related to cardiac arrest, and how we used LBD to gain insights into how these metabolites relate to cardiac arrest. These insights lead to our proposal (for the first time) of LCAT as a druggable target; the effects of which are supported by *in vivo* studies which were brought forth by this work. Metabolites are the end product of many biochemical pathways within the human body. Observed changes in metabolite levels are indicative of changes in these pathways, and provide valuable insights toward the cause, progression, and treatment of diseases. Following cardiac arrest, we observed changes in metabolite levels pre- and post-resuscitation. We used LBD to help discover diseases implicitly linked via these metabolites of interest. Results of LBD indicated a strong link between Fish Eye disease and cardiac arrest. Since fish eye disease is characterized by an LCAT deficiency, it began an investigation into the effects of LCAT and cardiac arrest survival. In the investigation, we found that decreased LCAT activity may increase cardiac arrest survival rates by increasing *ω*-3 polyunsaturated fatty acid availability in circulation. We verified the effects of *ω*-3 polyunsaturated fatty acids on increasing survival rate following cardiac arrest via *in vivo* with rat models.

## 1 Introduction

In this paper, we apply literature based discovery (LBD) to gain insights into the metabolic processes related to cardiac arrest and propose, and propose lecithin cholesterol acyltransferase (LCAT) as a druggable target for cardiac arrest. LBD aims to create computerized methods that support discovery from existing literature. LBD is typically performed by piecing together fragments of information in a meaningful way to support scientific discovery. Scientific publication is the primary means of disseminating scientific knowledge, and millions of scientific publications are stored in electronic databases with thousands more added each day (Bornmann and Mutz, 2015). The rate of scientific publication continues to grow (Hunter and Cohen, 2006), and this volume of data means that scientific knowledge is becoming fragmented, and pieces of related information may remain disjoint, even though their combination may lead to meaningful insight or scientific discovery. Integrating LBD into scientific research has the potential to transform the overwhelming amount scientific literature into a wellspring of new knowledge.

**FIGURE 1:**
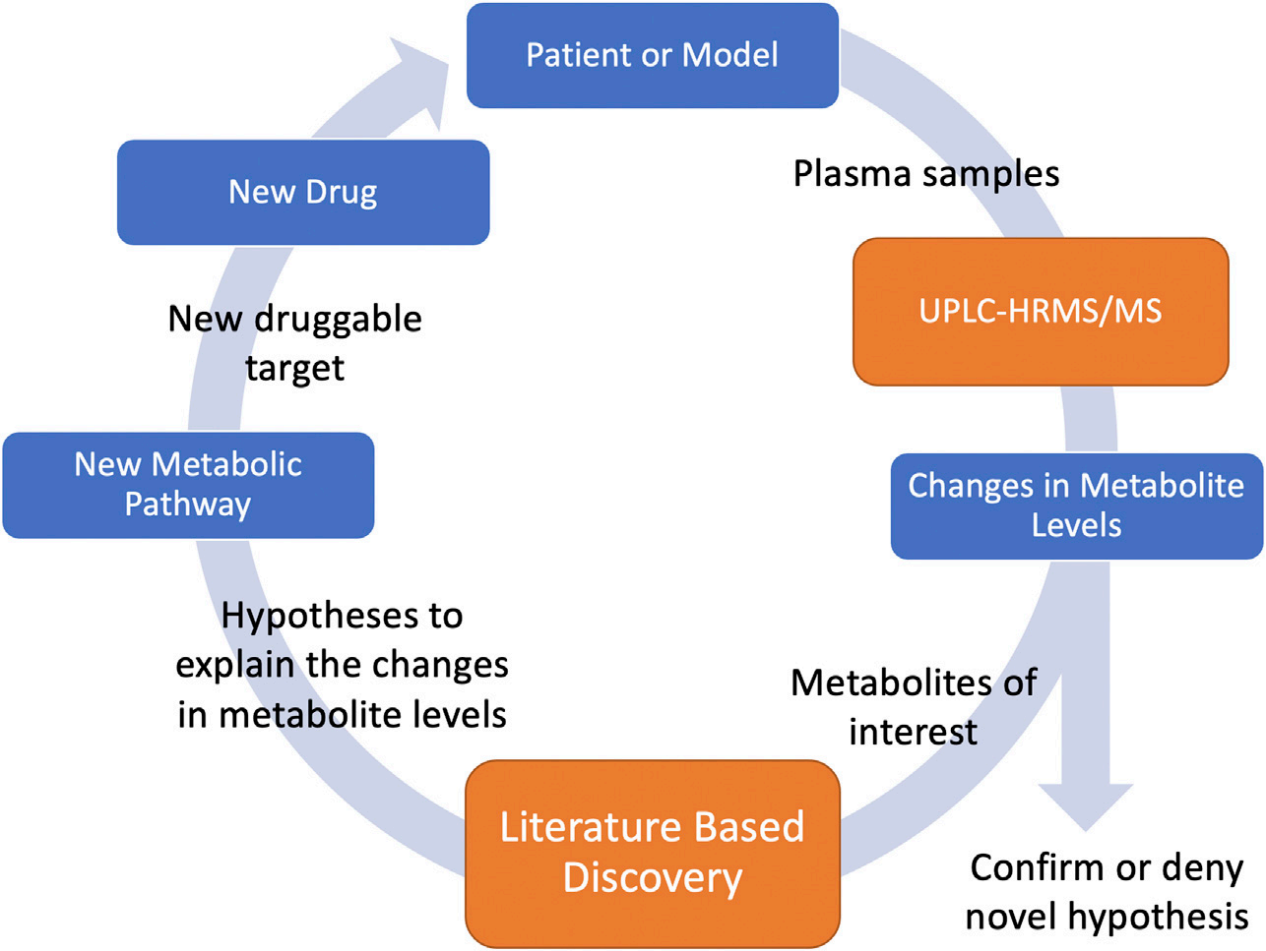
UPLC-HRMS/MS measures metabolite levels in plasma samples. Based on changes of metabolite levels, metabolites of interest are identified and input into LBD which generates hypotheses to help explain these observed changes. Based on these hypotheses, new metabolic pathways related to the disease of study are discovered. Druggable targets along the pathway can be identified and new drugs can be used to help treat the disease. Using these drugs, new plasma samples can be collected and the metabolic levels can be measured to support or deny the hypothesis.

Metabolomics is a field of study that utilizes the quantitative measurements of metabolites. Metabolites are the end results of many of the biochemical pathways that determine our health. Levels of metabolites in our body can give clues to understanding biochemical processes. Disorders in biochemical pathways can cause health problems, and modification of specific biochemical pathways can result in health benefits. We use ultra-high performance liquid chromatography coupled to high resolution tandem mass spectrometry (UPLC-HRMS/MS) to quantify the levels of a large variety of metabolites within an organism. Because UPLC-HRMS/MS screens for an enormous variety of metabolites, it effectively allows us to capture snapshots of an organism’s metabolome during the progression of a disease. Fluctuations in particular metabolite levels during disease progression indicate that the metabolite or the biochemical process that created it is associated with the disease. Unfortunately, these biochemical processes are extremely complex and gaining insights into their mechanisms can take years. LBD offers the potential to rapidly search huge amounts of scientific literature to generate novel hypotheses associating these metabolites to the disease pathophysiology. These hypotheses allow us infer novel metabolic pathways responsible for the observed fluctuations associated with the disease. Such knowledge allows us to identify novel druggable targets for the disease under investigation. Identification of druggable targets is the first step in drug development and drug repurposing, and therefore, combining LBD with metabolomics has the potential to massively decrease drug development time by quickly finding new druggable targets for specific diseases. Furthermore, we can predict the effect of these new drugs on the metabolome. We can then test the predictions by administering the drug, collecting new plasma samples, and undertaking additional metabolomic analysis. Confirmation of the predicted effects support the hypotheses about the metabolic processes and support the use of the drug to treat the disease. Unpredicted effects can lead to further inquiry supported by LBD, and the process can be repeated to gain increasing insight into the biochemical processes of the disease. This forms a feedback loop (Figure 1) where knowledge can be gained and multiple hypotheses tested iteratively until all knowledge that can be gained from the sample under examination is maximized.

In this work, we fully describe and expand upon our previous work related to metabolomic knowledge discovery. In our first exploratory investigation (Panahi et al., 2018), we briefly described the potential to use LBD for metabolomic research. In it, we stated that there is a potentially interesting, yet unexplored link between fish eye disease (also called partial lecithin cholesterol acyltransferase (LCAT) deficiency) and cardiac arrest, but excluded details and lacked any empirical evidence to support this hypothesis. Following that initial insight, we realized that LCAT and *ω*-3 share an underlying metabolic pathway. Since previous research suggested that *ω*-3 PUFA has some protective effects related to cardiac arrest, we were excited about this realization. In our next step (Cheng et al., 2020), we verified *in vivo* that *ω*-3 statistically significantly improved post-resuscitation myocardial dysfunction, which corresponds specifically to our collected data. We showed that the combination of *ω*-3 PUFA and ascorbic acid treatment confer an additive effect in suppressing lipid peroxidation thereby improving myocardial function and increasing survival rate following cardiac arrest. In this paper, we fully describe this process and validate the technique with empirical evidence showing that *ω*-3 PUFA vastly improves cardiac function leading to improved survival rates. Specifically, we fully describe our integration of LBD with ultra-high performance liquid chromatography coupled to high resolution tandem mass spectrometry (UPLC-HRMS/MS), fully describe our LBD methodology and results, propose LCAT as a druggable target for cardiac arrest and describe our reasoning behind it. We begin this paper with background information about metabolites and LBD before we present our particular methods including the resources used, how data was collected, and the LBD process. We then describe the LBD results, metabolomic findings, and *in vivo* verification. Lastly, we describe lessons learned in developing LBD for this application, limitations, future work, and conclusions.

## 2 Background

In this section we describe background information associated with our study.

### 2.1 Metabolites

The metabolome is the sum of all metabolites in an organism, and metabolites are small molecules that are the end result of many biochemical processes. Metabolites play a central role in maintaining the biochemical homeostasis which is critical for good health. Biochemical homeostasis is regulated in a process starting with DNA and ending with metabolites. DNA is transcribed into RNA which creates proteins. Many of the proteins are enzymes that catalyze the production and degradation of metabolites. Changes in an organism’s biochemical processes results in changes in the metabolome which can cause, and lead to the progression of diseases. Alternatively, pharmaceutically induced modifications to correct aberrant metabolic processes form the basis of disease treatment. Observing changes in a metabolite’s level can provide evidence that metabolite plays a role in contributing to a disease or recovery from it.

One important group of metabolites are lipids. Lipids are hydrophobic biomolecules such as fatty acids, glycerides, phospholipids, sterols, sphingolipids, and prenol lipids. The role of lipids in an organism include maintaining membrane structure, storage of energy, and providing energy for organs such as the heart. Lipids undertake a direct signaling role in an organism, such as in steroidal hormones, endocannabinoids, eicosanoids, ceramides, and platelet activating factor. The lipidome also affects signaling via the modulation of membrane curvature and fluidity. Being the external boundaries of cells and organs, lipids are directly exposed to the greater biochemical changes in a system, and undergo changes as a consequence. Examples include the oxidation and peroxidation of lipids under conditions of oxidative stress. As such, investigating the changes to the lipidome and the larger metabolome provide a window into the greater biochemical disruptions happening at a system level.

Since metabolites (and lipids in particular) play such a crucial role in the higher biochemical signaling levels which regulate biochemical processes, their metabolic pathways represent valuable drug targets. Lipids directly affect the metabolism of drugs which impacts a drug’s absorption rate and delivery mechanism. Multiple studies have demonstrated the relevance of the lipidome in nearly all diseases. A large number of FDA approved pharmaceutical interventions already exist for the modulation of lipid pathways ranging from the most common Aspirin (Patel and Baliga, 2020) to the highly efficacious and the most modern pharmaceutical interventions like monoclonal antibodies for the inhibition of PCSK-9 (Feinstein and Lloyd-Jones, 2016). Despite this, the large-scale investigation of the lipidome is relatively new indicating opportunities for impactful and actionable discoveries to be made with respect to human health and disease.

### 2.2 Cardiac Arrest and Metabolites

Cardiac arrest occurs when the heart suddenly stops beating due to a loss in the coordination of electric impulses that maintain the rhythmic contraction and relaxation of heart muscles. This causes a cessation of blood flow to major organs such as the brain. It is a major health problem which most often results in death (Khatib et al., 2017). It is one of the leading causes of death in the United States (Jazayeri and Emert, 2019), and even if patients initially survive, many fail to regain full functionality. Among those that survive, subsequent myocardial dysfunction are common. Additionally, ischemia reperfusion injury to major organs such as the brain often lead to neurological dysfunction and death (Benjamin et al., 2018). Economically, the cost of cardiac arrest is massive, and on average costs $3,750 per family per year tallying to a economic burden of $455 billion (Lurie et al., 2017).

Previous studies have shown that lipids play an important role during cardiac arrest. Disorders of lipid metabolism occur after global myocardial ischemia/reperfusion (I/R) injury (Siscovick et al., 2017; Xiao et al., 2020) which is a main cause of myocardial dysfunction after resuscitation (Patil et al., 2015). Furthermore, previous studies found that the lipid, *ω*-3 PUFA can reduce the risk of cardiac arrest (Leaf et al., 2003) as well as death from sudden cardiac arrest (Marchioli et al., 2002). Furthermore, because lipids are susceptible to oxidation, anti-oxidants such as ascorbic acid can increase the bio-availability of these molecules. Previous studies have shown that the administration of the ascorbic acid at the start of cardiopulmonary resuscitation (CPR) decreased myocardial damage and improved survival rate and neurological outcome in a rat model of cardiac arrest and CPR (Tsai et al., 2011, 2014). Ascorbic acid is an effective anti-oxidant, and current evidence indicates that the combination of two or more antioxidants may exert synergistic myocardial protective effects (Satyanarayanan et al., 2018; Vineetha et al., 2018). Although these studies indicated that *ω*-3 PUFA and ascorbic acid played some role in cardiac arrest, no studies prior to this research showed the effects of *ω*-3 PUFA and ascorbic acid on the mechanisms of post-resuscitation myocardial dysfunction when given acutely following cardiac arrest. Furthermore, no studied established the link between LCAT, *ω*-3 PUFA, and cardiac arrest.

### 2.3 Ultraperformance Liquid Chromatography–High-Resolution Mass Spectrometry Mass

We use ultra-high performance liquid chromatography coupled to high resolution tandem mass spectrometry (UPLC-HRMS/MS), which is a multipurpose analytical tool to quantitatively and qualitatively analyze biomolecules. This tool first separates biomolecules by their chemical properties (UPLC) and then by their mass to charge ratio (m/z). The combined techniques allows for a very high level of unambiguous identification of molecules at a sensitivity that surpasses a majority of other analytical technologies. The molecular identity is confirmed via a combination of their retention time, intact molecular m/z as well as the m/z’s of the fragments of a specific molecule. The combination of these parameters in a majority of cases are unique to a particular molecule thereby allowing us to assign a molecular identity by comparing them against a database of known molecular parameters. Where the identity of the molecule has not been reported previously, the information obtained still allows us to hypothesize the possible structure of the molecule, and thereby assign a putative identity that can later be confirmed via comparison against an authentic standard or via an orthogonal method such as nuclear magnetic resonance (NMR). All in all, UPLC-HRMS/MS is the most versatile tool available to date for investigating biochemical changes associated with health and disease.

### 2.4 Literature Based Discovery

Literature based discovery (LBD) was first conceptualized by Dr Don Swanson, who formalized his approach of finding implicit links in the literature to discover new knowledge. In his seminal work (Swanson, 1986), he discovered a previously unknown link between Raynaud’s disease and Fish Oil through their interactions with several intermediate terms, primarily blood viscosity, platelet aggregation, and vascular reactivity (Weeber et al., 2001). This link was found through an analysis of existing literature and was later verified via clinical trials (DiGiacomo et al., 1989), which gave credence to the idea that vast amounts of undiscovered knowledge lay hidden in scientific literature. Since that initial discovery, LBD techniques have facilitated knowledge discovery related to cataracts (Kostoff, 2008b), multiple sclerosis (Kostoff et al., 2008b), and Parkinson’s disease (Kostoff and Briggs, 2008). LBD has led to understanding and discovering new health benefits of curcumin (Srinivasan and Libbus, 2004) and discovering potential treatments for cancer (Ahlers et al., 2007; Zhang et al., 2014).

The primary application areas of LBD have been for drug development (Hu et al., 2003; Hristovski et al., 2010; Zhang et al., 2014), drug repurposing (Ahlers et al., 2007; Baker, 2010; Deftereos et al., 2011; Cohen et al., 2014; Zhang et al., 2014; Rastegar-Mojarad et al., 2015, 2016; Yang et al., 2017; Zhang et al., 2020), and adverse drug event prediction (Deftereos et al., 2011; Banerjee et al., 2014; Shang et al., 2014; Hristovski et al., 2016; Mower et al., 2016). Although we were unable to find prior studies focused specifically on applying LBD to metabolomic knowledge discovery, biochemical pathways are frequently an area of investigation for new drug development. Gubiani et al. (2015) used the previously developed RaJoLink LBD system (Petrič et al., 2009) to support biomedical discoveries related to aging. Their goal was to validate links found between gut bacteria and aging. They mention that these links have been found by combining genetic sequencing approaches, proteomic studies, and metabolomic studies. Hansson et al. (2020) described a method to support novel drug discovery related to diabetes. Their focus was to investigate the effects of proteins on different metabolic pathways. They developed a system to find evidence that supports drug discovery using explicit connections “right after publication” rather than finding implicit connections across all literatures as is traditionally the case with LBD. Much related work had focused on genetic data (Hu et al., 2003; Frijters et al., 2010; Hristovski et al., 2010; Faro et al., 2012; Zhang et al., 2014). In one study, Frijters et al. (2010) used LBD to investigate the connections between genes, drugs, biological pathways, and diseases. Their study included an *in vitro* verification of their proposed connection between damnacanthal’s and dephostatin’s inhibition of cell proliferation. In another study, Faro et al. (2012) integrated LBD with genetic data (such as micro-arrays), and used it to study genetic pathways and gene regulation. They described their tool, GeneWizard, which served as an aid in discovering new gene-disease relations.

Applications of LBD outside of the biomedical domain include developing efficient water purification systems (Kostoff et al., 2008d), accelerating the development of developing countries (Gordon and Awad, 2008), categorizing potential bio-warfare agents (Swanson et al., 2001), studying climate change (Aamot, 2014), and identifying promising research collaborations (Hristovski et al., 2015).

In the traditional ABC co-occurrence model (Swanson and Smalheiser, 1997) of LBD, a start (*A*) term is inputted into the system. Text is searched to find the set of all terms that co-occur with the *A* term, forming the set of linking (*B*) terms. Again, the text is searched but this time the set of all terms that each *B* term co-occurs with are found, forming the set of target (*C*) terms. In the obligatory example of Swanson’s Raynaud’s Disease - Fish Oil discovery, Raynaud’s disease is the start term, blood viscosity, platelet aggregation, and vascular reactivity are the linking terms, and fish oil is the target term.

Using this model, there are two primary modes of LBD: closed-discovery and open discovery (Weeber et al., 2001) [also called two-node search and one-node search respectively (Swanson and Smalheiser, 1997)]. In closed discovery, the goal of LBD is to help explain a hypothesized connection between the start and target term. The end result is a set of linking terms which describe how the start and target term are related (e.g. blood viscosity, platelet aggregation, and vascular reactivity). In open discovery, LBD helps to find new concepts implicitly linked to the start term. These new connections can provide novel insights such as treatments for a disease or symptoms (e.g. fish oil). These two paradigms are not exclusionary, and hypotheses generated using open discovery can be explained using hypotheses generated from closed discovery. Furthermore, in either case the end goal of LBD is the same - to generate hypotheses from knowledge implicit in literature.

**FIGURE 2:**
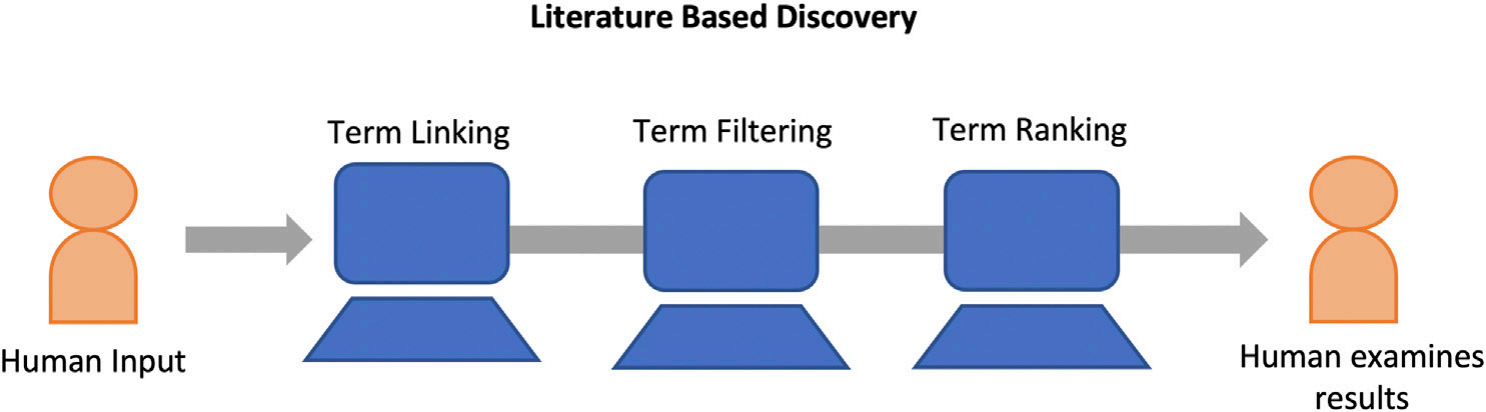
LBD systems typically consist of at least three components. Term linking, term filtering, and term ranking. Hypotheses are generated (term linking), spurious hypotheses are removed (term filtering), and the remaining hypotheses are ranked (term ranking) and displayed to the user for analysis.

Presently, LBD is a mature field with varying paradigms and system designs (Henry and McInnes, 2017; Kastrin and Hristovski, 2020). Despite this variation, at their most basic, most LBD systems share a common process consisting of at least three primary steps: 1) Term Linking, 2) Term Filtering, and 3) Term Ranking. Figure 2 provides a high level view of the LBD process. Here, we discuss each of these steps in more detail as well as current LBD evaluation methodologies.

#### 2.4.1 Term Linking

The initial *A* to *B* and *B* to *C* relationships are identified based on explicit relationships in text. These relationships may be based on co-occurrences (Swanson and Smalheiser, 1997; Weeber et al., 2001; Yetisgen-Yildiz and Pratt, 2006), semantics (Hristovski et al., 2006; Preiss et al., 2015; Rastegar-Mojarad et al., 2015), vector operations (Gordon and Dumais, 1998; Bruza et al., 2004; Cohen et al., 2010, 2011), or other methods (Kostoff et al., 2008c; Goodwin et al., 2012; Workman et al., 2016).

#### 2.4.2 Term Filtering

Term linking tends to over-generate linking and target terms, and during the term filtering step, terms that are already known, too obvious, spurious, or otherwise uninformative are removed. Term filtering techniques are commonly frequency-based, semantic-based, or relation-based. Frequency-based filters (Gordon and Lindsay, 1996; Pratt and Yetisgen-Yildiz, 2003; Preiss et al., 2015) remove terms by thresholding based on some count, statistical measure, or cosine distance between vector representations. These are effective for removing terms that are too common or uncommon. Semantic type filters (Weeber et al., 2001) restrict or remove terms based on their meaning. Typically this is performed using dictionaries in which terms are categorized into various semantic types which broadly characterize the words meaning. For example, target terms may be restricted to only “Drugs” if the user is searching for a new treatment for a disease. Relation type filters (Hristovski et al., 2006) remove terms based on how they are related to each other. If terms are semantically linked using relationship extraction, it is often possible to label the type of relation between the two terms. Relation type filters restrict or remove terms based on the labels of these relations. For example, we may want to restrict the start and linking terms to “Affects”, “Regulates”, “Increases”, or “Decreases” type relationships to discover how a drug and disease interact via a set of linking terms. Other types of filters exist, but are less common. One example is hierarchical-based filters (Pratt and Yetisgen-Yildiz, 2003; Hu et al., 2006) which attempt to eliminate terms that are too general based on their distance from the root of semantic hierarchies.

#### 2.4.3 Term Ranking

During term ranking, hypotheses are ranked based on some measure of interestingness. Even after term filtering, there may remain too many hypotheses to manually review and term ranking helps to prioritize the user’s analysis. Linking Term Count (LTC) (Swanson and Smalheiser, 1997) is one of the first developed and best performing (Yetisgen-Yildiz and Pratt, 2009; Henry and McInnes, 2019) ranking measures. LTC ranks each target term by counting the number of unique linking (*B*) terms between the start (*A*) and target (*C*) term. However, there are a huge variety of ranking methods (Henry and McInnes, 2017). These include co-occurrence measures (Gordon and Lindsay, 1996; Swanson and Smalheiser, 1997; Hristovski et al., 2001, 2005; Swanson et al., 2006), statistical measures (Wren, 2004; Yetisgen-Yildiz and Pratt, 2009; Rastegar-Mojarad et al., 2015; Henry and McInnes, 2019), vector-based measures (Gordon and Dumais, 1998; Bruza et al., 2004), and graph-based measures (Wilkowski et al., 2011; Eronen and Toivonen, 2012).

#### 2.4.4 Literature Based Discovery Evaluation

There are a few standard evaluation methodologies for LBD: discovery replication, time-slicing (Yetisgen-Yildiz and Pratt, 2009), and link prediction (Eronen and Toivonen, 2012; Kastrin et al., 2016). For discovery replication, past discoveries are remade using similar data to that available at the time of the original discovery. Time slicing assesses a system’s ability to generate new discoveries by splitting a dataset chronologically into training and test sets; potential discoveries are generated on the training set and their existence is validated on the test set. Similarly, link prediction assesses a system’s ability to generate new discoveries by splitting a knowledge graph into training and test sets. *True* edges (links) are removed from the graph and are combined with *false* edges which do not exist on the graph. Performance is evaluated by a system’s ability to distinguishing between these true and false edges.

These standard evaluation methodologies aid in system development, but the ultimate goal of LBD is to generate practical new knowledge, and a system’s ability to do so is the best indicator of a system’s success. While LBD researchers often claim new discoveries, many of these discoveries have failed to withstand empirical evaluation and expert assessment (Bekhuis, 2006; Kostoff, 2008a; Kostoff, 2008c; Kostoff et al., 2008a). Therefore, combining new discoveries with empirical evaluation is essential. Examples of empirical evaluation include evidence from microarray data (Hristovski et al., 2010) and proteomic data (Hu et al., 2003), *in vitro* testing (Frijters et al., 2010; Cohen et al., 2014), *in vivo* testing (Wren et al., 2004; Lekka et al., 2012), and clinical trials (DiGiacomo et al., 1989). Furthermore, a shortcoming of LBD has been its relative lack of adoption in its intended domain. Generating discoveries with LBD and validating with empirical evidence exposes LBD to new audiences and gives credibility to the field. Working with domain experts to validate discoveries sparks collaborations between LBD developers and users which will lead to more useful LBD software in future iterations.

## 3 Materials and Methods

In this section we describe the materials and methods utilized in our study.

### 3.1 Materials

#### 3.1.1 Unified Medical Language System

The Unified Medical Language System (UMLS) is a knowledge representation framework designed to support biomedical and clinical research. It is a data warehouse containing three knowledge sources: Metathesaurus, Semantic Network and SPECIALIST Lexicon. The Metathesaurus is a multi-lingual lexical database that combines information about biomedical and health-related concepts from various biomedical and clinical sources (e.g. Medical Subject Headings). It includes over 100 knowledge sources and classification systems encoded with different semantic and syntactic structures. The Metathesaurus organizes knowledge based on Concept Unique Identifiers (CUIs). The CUIs are further categorized into 127 semantic types and 15 semantic groups.^1^ For example, the concept “Cardiac Arrest” has a CUI C0018790 and is semantic type “disease or Syndrome (T047)” which belongs to the semantic group “Disorders (DISO)”.

#### 3.1.2 MetaMapped MEDLINE Baseline

In this work, we utilize the National Institutes of Health (NIH), National Library of Medicine’s (NLM’s) MetaMapped MEDLINE baseline.^2^ MEDLINE contains over 20 million biomedical and clinical citations from 1966 to present day. MetaMap (Aronson, 2001) is a concept mapping system that maps terms in biomedical text to CUIs in the UMLS Metathesaurus. The titles and abstracts from MEDLINE citations are periodically processed by MetaMap and released via the MetaMapped MEDLINE baseline.^3^. For this study, we used the 2015 version of the MetaMapped MEDLINE baseline.

#### 3.1.3 Data Collection

To identify the metabolites associated with cardiac arrest, plasma samples (*n* = 28) were collected from cardiac arrest patients at time of arrival to the hospital post resuscitation and following therapeutic hypothermia target body temperature. Metabolites were extracted from these samples followed by analysis with untargeted lipidomic and metabolomic approaches. The resultant data were normalized and the top most statistically significant metabolites were identified. The study was undertaken using residual samples from a study already approved by the Institutional Review Board (IRB protocol number: HM15326). Only the metabolomic and lipidomic data obtained from the study was used in this manuscript.

Plasma from cardiac arrest patients were analyzed via UPLC-HRM/MS and the abundance of all identifiable species were collected. Thereafter the species abundances were analyzed statistically and those which were significantly different (p ≤ 0.05) were identified for further investigation. Through this process, we identified 22 metabolites that passed our statistical criteria as being of being significant with respect to the post cardiac arrest period. These counts are indicative of changes in biochemical pathways of which the metabolites are the end product.

Of the 22 identified metabolites, 19 mapped to UMLS CUIs and three metabolites did not have associated UMLS CUIs (4-acetamidobutyric acid, Lysophosphocholine, and N(epsilon) Methyl-l-lysine). Table 1 shows UMLS CUIs and their preferred term for each of the *B*-Terms and the initial *A*-term (Cardiac Arrest). During the manual concept mapping process, four metabolites mapped to two synonymous CUIs. In these cases, a single term was selected. Selection was based on the “preferred term” indicated by the UMLS. These include the following terms for which the selected concept used in LBD is shown in italics *C0019602 histidine* and C0523697 Histidine measurement, *C0023401 leucine* and C0428209 Leucine measurement, *C0003765 arginine* and C0523503 Arginine measurement, and *C0556150 docosahexaenoic acid* and C0012968 Docosahexaenoic acids. Two concepts never occurred with C0018790 Cardiac Arrest. As such, they could not be considered linking terms (since an *A* to *B* link does not exist). These concepts include C2348307 Docosadienoic acid and C0069409 oleoylcarnitine. The result is a set of 17 metabolites which co-occur with C0018790 Cardiac Arrest and are used to calculate ranking scores.

**TABLE 1:** Metabolites used in this study and their corresponding CUIs.

	CUI	Preferred term
A-term	C0018790	Cardiac arrest
B-terms used	C0368608	Acylcarnitine
	C0003765	Arginine
	C0007745	Ceramides
	C0008405	Choline
	C0556150	Docosahexaenoic acid
	C0058624	Docosapentaenoic acid
	C2348386	Eicosadienoic acid
	C2348388	Eicosatrienoic acid
	C0017770	Glucosylceramides
	C0019602	Histidine
	C0023401	Leucine
	C0024360	Lysophosphatidylcholines
	C0028375	Norleucine
	C0070662	Phenylalanylphenylalanine
	C0031716	Phosphorylcholine
	C0031951	Pipecolic acid
	C0037906	Sphingomyelins
No Co-occurrence with a term	C2348307	Docosadienoic acid
	C0069409	Oleoylcarnitine
No CUI mapping	-	4-Acetamidobutyric acid
	-	Lysophosphocholine
	-	N(epsilon) Methyl-l-lysine

### 3.2 Method

**FIGURE 3:**
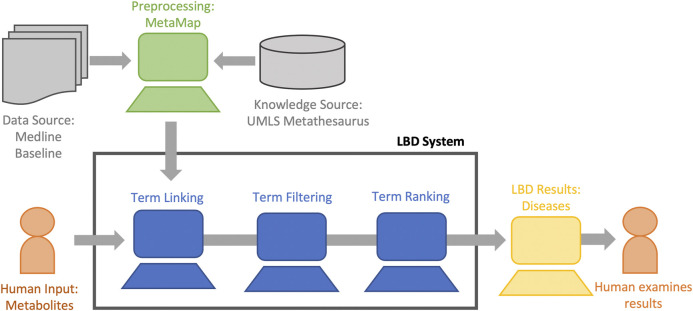
The overall LBD process of our system. Hypotheses are generated by finding relations implicit to the concept of interest (human input) using co-occurrence information from the data source. The hypotheses are filtered, ranked, and displayed to the user for analysis.

Figure 3 describes our LBD system at a high level (Henry and McInnes, 2019)^4^. Our system is an ABC co-occurrence based system based on text mapped to CUIs within the UMLS. The goal of our system is to find diseases highly associated with the metabolites we identified in the laboratory setting as relevant to cardiac arrest. Therefore, we input the start (*A*) term of cardiac arrest (C0018790), we restrict our linking (*B*) terms to the 19 metabolites of interest (described in Table 1), and restrict our output (*C*) terms to diseases. These diseases are then ranked and displayed to the user. We describe each step in our system in detail below.

**Preprocessing:** We extract co-occurrence information between concepts from the MetaMapped MEDLINE baseline. Due to the data being processed by MetaMap, stopwords are automatically removed as they do not map to UMLS CUIs, and synonymous terms are mapped to a single concept which can create a more accurate co-occurrence matrix. We construct a co-occurrence matrix using using a symmetric window size of eight, meaning that concepts are counted as co-occurring if they occur within eight concepts before or after the term the window is focused on. Sentence boundaries are ignored. A window size of eight was chosen because it is the average CUI length of sentences in the 2015 MetaMapped MEDLINE baseline. Furthermore, evidence from previous studies focused on semantic relatedness (Henry et al., 2018) found that a window size of eight does a good job at balancing between noise introduced by too large a window, and missing information caused by too small a window size. The end result is a flat file of co-occurrence counts of CUI pairs extracted from MEDLINE. The co-occurrence counts in the file are used in the term linking, term filtering, and term ranking steps of LBD.

**Term Linking:** The initial *A* to *B* and *B* to *C* relationships are identified based on explicit relationships using the collected co-occurrence information. The co-occurrence of terms in text constitutes a relationship; and the linking terms are found iteratively through co-occurrences. As noted above, we restrict our *B* terms to the 19 metabolites of interest summarized in Table 1.

**Term Filtering:** Uninformative terms are removed by applying a co-occurrence-count-based filter to remove any concepts with just a single co-occurrence. We also apply a semantic type filter which restricts the *C* terms to Diseases (semantic type: “T047 DYSN”).

**Term Ranking:** For LBD, it is assumed that target terms never co-occur with the start term (since they are new knowledge), meaning that traditional information retrieval ranking methods, which require direct co-occurrences cannot be applied. Instead, indirect ranking measures must be used. As previous work has shown LTC (Swanson and Smalheiser, 1997) is simple and effective (Yetisgen-Yildiz and Pratt, 2009; Henry and McInnes, 2019), we used LTC.

**Hypothesis Display:** The resultant data were provided to the researchers for interpretation and analysis. Data was provided as a list of ranked terms. A complimentary file was provided containing the ranked target terms and beneath them the target term’s co-occurrence counts with each of the linking terms. This allowed the researcher to quickly get an idea as to how (via what metabolites) the target term was related to the start term. Table 2 shows an example of this output.

**TABLE 2:** Example output showing the third and fourth highest ranked terms.

15 - C0342895 - disease, fish-eye		
503	C0556150	Docosahexaenoic acid
481	C0008405	Cholines
411	C0023401	Leucine
395	C0003765	Arginine
158	C0019602	Histidine
55	C0037906	Sphingomyelin
50	C0007745	Ceramide
29	C0368608	Acylcarnitines
19	C0058624	Docosapentaenoic acid
17	C2348388	Eicosatrienoic acid
15	C0031716	Phosphorylcholine
12	C0024360	Lysophosphatidylcholine
6	C0017770	Glucosylceramide
4	C0028375	Norleucine
3	C2348386	Eicosadienoic acid
**14 - C0043194 - aldrich syndrome wiskott**		
508	C0019602	Histidine
189	C0003765	Arginine
139	C0023401	Leucine
90	C0008405	Cholines
43	C0007745	Ceramide
35	C0031716	Phosphorylcholine
29	C0037906	Sphingomyelin
28	C0024360	Lysophosphatidylcholine
23	C0556150	Docosahexaenoic acid
6	C0368608	Acylcarnitines
6	C0017770	Glucosylceramide
2	C0028375	Norleucine
2	C0070662	Phenylalanylphenylalanine
2	C0058624	Docosapentaenoic acid

Although it is common to remove terms that directly co-occur with the starting term from LBD output, we chose to keep those terms and display them to the user. We have several reasons for this: First, Due to the volume of scientific literature, it is unlikely that an investigator is aware of all previously published studies. Hristovski et al. (2013) found that one of the most useful uses of LBD systems is to find information that is new to the investigator rather than new to science. Therefore, by removing directly co-occurring terms we remove information that is potentially meaningful to the investigator. Second, co-occurrences do not necessarily constitute a relationship. This is particularly true for terms that directly co-occur with the start term just a few times. Removing terms that directly co-occur with the start term may remove meaningful information. Third, even when terms didn’t provide any new information to the investigator (the term directly co-occured with the start term and was already known to the investigator), they helped to build trust in the efficacy of the LBD system. Investigators may be unfamiliar with LBD techniques and practitioners of traditional experiment-driven science may be skeptical of data-driven science such as LBD. By including these “known” terms in LBD output, it provides a level of assurance to the investigator that the rankings of terms are actually valid and meaningful. Lastly, due to differences in how ranking occurs for LBD ranking methods and traditional information retrieval ranking methods, we found that the top ranked LBD output contained minimal directly co-occurring terms, and that investigators could quickly skip over terms that provided no new knowledge to them with minimal impact on actual investigation time.

## 4 Results

In this section we describe our LBD and metabolomic results. The LBD results described here provided the spark of insight which began a line of inquiry into the relationship between LCAT and cardiac arrest. This inquiry led to the realization that LCAT has an effect on *ω*-3 PUFA availability. This, coupled with the association between several PUFAs and cardiac arrest found via plasma sample analysis and LBD further supported the potential of LCAT as a drug target for increasing cardiac arrest survival rates. Furthermore, previous literature (Marchioli et al., 2002; Leaf et al., 2003) has shown *ω*-3 PUFA’s protective effects against cardiac arrest, however no studies had shown whether an acute infusion of *ω*-3 PUFA post cardiac arrest improves myocardial dysfunction. Therefore as the next step, we demonstrated that *ω*-3 polyunsaturated fatty acids can indeed improve myocardial function thereby increasing survival rate following cardiac arrest, which indicates clinical relevance (Cheng et al., 2020). This was shown *in vivo* via rat models.

### 4.1 Analysis of Literature Based Discovery Results

Table 2 shows an example of the output of our LBD system. It shows the third and fourth highest ranked terms. The first highest ranked term was the start term, “C0018790 - Cardiac Arrest”. This was expected, however we found it was important to leave it in the list of ranked terms because it supports the validity of this technique. Furthermore, since it co-occurs with only 17 of the 19 *B* terms, it indicates that two of the metabolites (docosadienoic acid and oleoylcarnitine) we identified via UPLC-HRMS/MS had never been reported with Cardiac Arrest. This indicates a potentially new association worthy of investigation. The second highest ranked term was “C0012634 - disease, NOS” which is too general a term to provide any meaningful insight. Our system outputs hypotheses as a ranked list of target concepts and their co-occurrence frequencies with the pre-defined set of *B* terms. In the output, the target term appears and below it (and tab indented) is the list of *B*-terms and the count of *B* to *C* co-occurrences. This was done to facilitate an understanding of how the disease is related to the start term (Cardiac Arrest) at a glance. The *B*-terms are listed in descending order of co-occurrence. No tie-breaking method was used when determining which terms to display first for either the *C*-terms or the *B*-terms. Instead, their appearance is purely by chance.

Interpreting Table 2, the first row tells us that “C0342895 - disease, fish eye” co-occurs with 15 of the 19 identified metabolites. Below this, these 15 metabolites are listed in descending order, ranked by the count of co-occurrences with each of the metabolites. The numbers tell us that “C0556150 - docosahexaenoic acid” co-occurs with fish eye disease a total of 503 times. This frequency of co-occurrence indicates the relative strength of the relationship between docosahexaenoic acid and fish eye disease. Next “C0008405 - cholines” co-occurs with fish eye disease 481 times. Each co-occurring metabolite is listed until lastly “C2348386 - Eicosadienoic acid” is listed, which co-occurs with fish eye disease just three times, indicating a relatively weak relation between itself and fish eye disease. The rankings of the metabolites provide clues as to what relation to begin investigating. The link between fish eye disease, docosahexaenoic acid (an *ω*-3 PUFA), and cardiac arrest is clearly indicated in this table and led to the starting point of discovery.

The next highest ranked term in our LBD output was “C0043194 - Wiskott Aldrich syndrome”, which co-occurs with 14 of the 19 identified metabolites. The frequency of co-occurrence between it and each metabolite is listed below it. For “C0043194 - Wiskott Aldrich syndrome”, we see that different metabolites co-occur with it more frequently, indicating that it is more strongly related to metabolites different than those strongest related to fish eye disease. We have not yet performed an investigation into Wiskott-Aldrich syndrome, however it may be of interest for future work.

Table 3 shows a histogram of the number of terms per LTC score. As summarized in Table 3, there were 55,376 disease terms identified. Of them, only 3,122 co-occur with any of the linking terms; 553 occur with two or more linking terms; 501 with three or more; 306 with four or more; 195 with five or more; 126 with six or more; and 88 with seven or more, and so on. Note the Zipfian distribution of the histogram. With less than 100 terms, this resulted in a manageable number for manual review. Of course, restricting to *B* terms of interest requires a lot of forethought and knowledge of the problem, however it is often the case that researchers know what they are looking (at least at a high level) before inquiry begins.

**TABLE 3:** Histogram of the number of terms per LTC score.

LTC	Target terms with this LTC score	Count of terms with >= LTC
17	1	1
16	1	2
15	1	3
14	1	4
13	1	5
12	1	6
11	4	10
10	11	21
9	28	49
8	15	64
7	24	88
6	38	126
5	69	195
4	111	306
3	195	501
2	452	953
1	2,169	3,122
0	52,254	55,376

A large portion of the top ranked terms were too general to be of interest (e.g. Diseases, vesicle, communicable diseases). Although we performed only a manual review process, it is possible that term filtering methods that take advantage of the UMLS hierarchy could be useful in future work. The prevalence of these uninformative terms, however made the informative ones all that more interesting.

Terms with an LTC of 10 or more produces a very concise list of only 21 terms as shown in Table 4. A manual review of these terms shows that six are too general to provide much valuable insight. So, in reality only 15 of the terms provided specific information for a researcher. A complete set of the rankings and results displayed are available online.^5^

**TABLE 4:** List of terms with an LTC of 10 or greater.

LTC	CUI	Preferred term
17	C0018790	Cardiac arrest
16	C0012634	Disease, NOS
15	C0342895	Disease, fish-eye
14	C0043194	Wiskott aldrich syndrome
13	C0162429	Malnutrition NOS
12	C0028754	Obesity, NOS
11	C0333262	Vesicle (morphologic abnormality)
11	C0009450	Communicable disease, NOS
11	C0034341	Deficiency disease, pyruvate carboxylase
11	C0011860	Diabetes mellitus, non insulin dependent
10	C0010054	Arteriosclerosis, coronary
10	C0243026	Sepsis, NOS
10	C1720830	Painful bladder syndrome
10	C0025517	Metabolic disease, NOS
10	C0002395	alzheimer’s diseases
10	C0036690	Septicaemia, NOS
10	C0007222	Cardiovascular disease, NOS
10	C0026769	Multiple sclerosis, NOS
10	C0011389	Dental plaques
10	C0039082	Syndrome, NOS
10	C0175697	Van der woude’s syndrome

#### 4.1.1 Disease Findings

Among the diseases, shown in Table 4, Fish Eye disease ranked high with 15 of the metabolites of interest being associated with the disease. Fish eye disease, also called partial lecithin cholesterol acyltransferase (LCAT) deficiency is caused by mutations in the LCAT gene. This mutation reduces LCAT’s ability to remove cholesterol from the blood. Very little literary evidence was found for a direct relationship between LCAT and cardiac arrest and the mechanisms as to how it may be related were unknown. Fish eye disease’s relation to LCAT, however gave a clear pathway toward further investigation.

#### 4.1.2 Metabolite Findings

Table 5 shows the 19 metabolites identified as the most significant metabolic differences between arrival and target body temperature. 17 of the 19 target metabolites were identified as co-occurring with previous studies with cardiac arrest. Recall that these 19 metabolites were observed to have statistically significant changes post-resuscitation following cardiac arrest via our plasma sample collection and subsequent analysis. This LBD approach allows for a computational approach to verify data collection and analysis, rather than an arbitrary approach. Furthermore, two metabolites, docosadienoic acid and oleoylcarnitine had never before been reported in the context of cardiac arrest indicating a potentially new association.

**TABLE 5:** Significant metabolites identified.

Phosphocholine	Ceramide
Histidine	Acylcarnitine
Sphingomyelin	Lysophosphocholine
Phenylalanylphenylalanine	Docosapentaenoic acid
Glucosylceramide	Leucine
Docosadienoic acid	Choline
Pipecolic acid	Oleoyl l-carnitine
Arginine	*Docosahexaenoic acid
Eicosatrienoic acid	*Eicosadienoic acid
L-norleucine	

### 4.2 Metabolic Mechanisms

**FIGURE 4:**
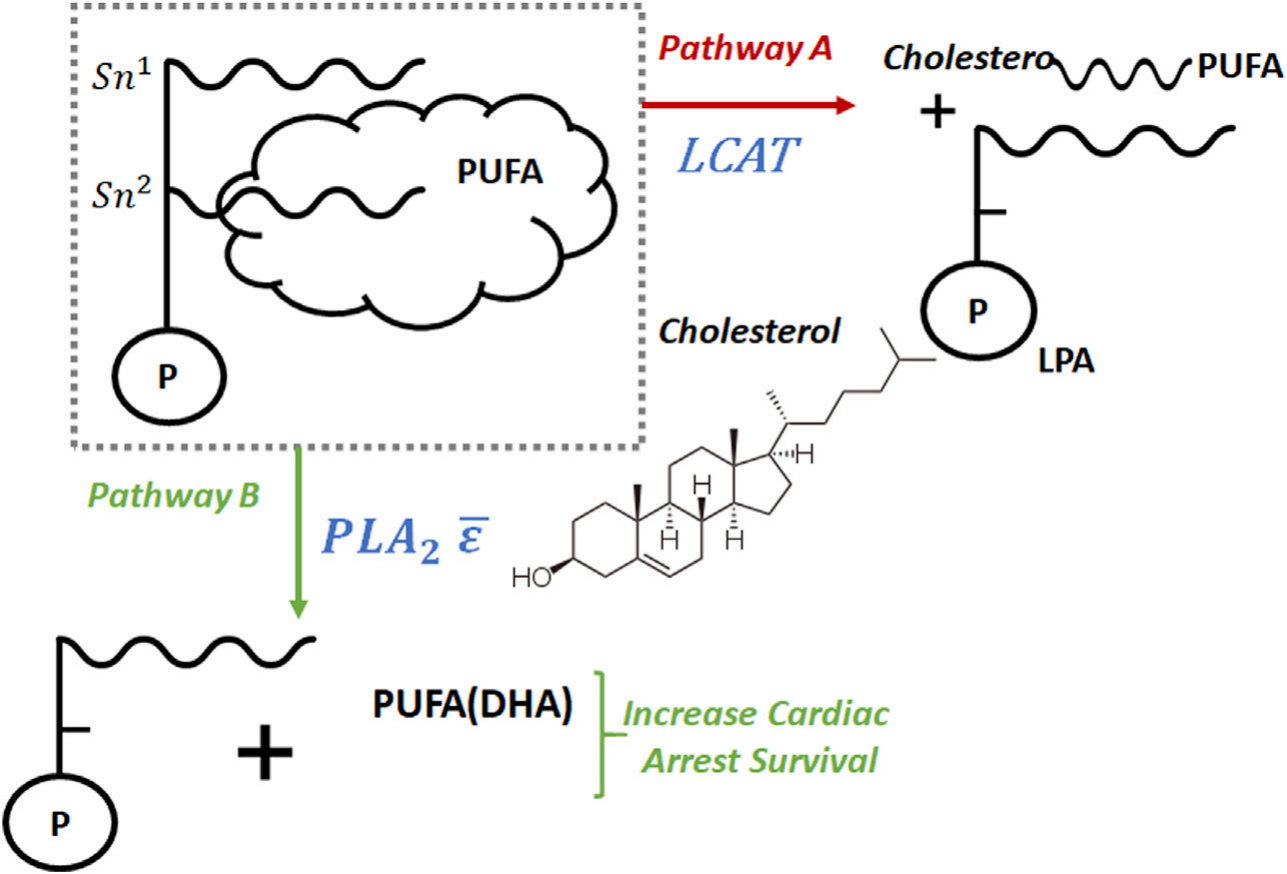
If LCAT increases we expect a lower amount of PUFA(DHA) through pathway B and an increases amount of cholesterol esters containing PUFA and LPA through pathway A, which results in decrease of cardiac arrest survival.

The coupled metabolic and disease LBD findings provided the spark of insight which began a line of inquiry into the connection between LCAT and cardiac arrest via its effect on PUFA availability. Figure 4 shows the connection between LCAT and PUFA. In it, we show two competing pathways. Pathway A is regulated by LCAT, and in it, cholesterol esters containing PUFA and LPA are formed resulting in PUFA being stored in tissue, thereby removing it from circulation. Alternatively, in Pathway B, PUFA (including DHA) remains in circulation, making it available to increase cardiac arrest survival rates. We can think of these pathways as a split in a river. Pathway A removes PUFA from the system, and pathway B makes it available. By decreasing the flow of PUFA into pathway A, we increase its flow into pathway B, thereby increasing PUFA availability in the system. LCAT regulates pathway A, and by decreasing LCAT availability we decrease PUFA flow into pathway A resulting in increased cardiac arrest survival.

The disorders of lipid metabolism in the heart occur following resuscitation. Supplementation with *ω*-3 PUFA had previously shown to reduce the risk of cardiac arrest however the effects of the *ω*-3 PUFA and mechanisms post resuscitation myocardial dysfunction had not been investigated. Under normal conditions, LCAT transfers polyunsaturated fatty acids (PUFA) from the Sn-2 position of phospholipids to cholesterol in order to generate cholesterol esters (CE). Once transformed in this manner, CE is removed from circulation and goes into storage in tissue. In effect this mechanism removes both cholesterol and PUFA from the system. Previous studies by us (Xiao et al., 2020) have demonstrated that docosahexaenoic acid (DHA), a PUFA with 22 carbons and six double bonds are associated with survival following cardiac arrest. Figure 4 shows a high level view of the metabolic pathways. The data obtained via this LBD exercise demonstrated the activation of the LCAT pathway which removes required DHA from the system at a time where it is most needed.

Subsequent rat model experimentation following these insights demonstrated that artificially increasing the DHA content via infusion following cardiac arrest in a rat model led to statistically significant improvement in cardiac function. The investigation led to identifying that either ω-3 PUFA or ascorbic acid at the start of a cardiac arrest can significantly alleviate inflammation, oxidation stress, and myocardial injury which contributes to enhanced cardio-protections. However their combination provides better protection of myocardial function due to the alleviation of lipid peroxidation by the synergy effect of the combination (Cheng et al., 2020).

## 5 Discussion

The application of LBD to metabolomics showed that it can provide hitherto unknown drug targets for treating diseases, such as modulating LCAT for treating post cardiac arrest syndrome. Similar high ranking diseases (e.g. diabetes, septicemia) were also found to have high levels of relationship to cardiac arrest via their common metabolites, confirming the presence of long suspected, but never before proven, common underlying metabolic circuits between these different diseases. The practical applications of such findings include the ability to generalize insights gained and treatments devised for one disease to others that are closely linked metabolically leading to faster translation of bench-side research to clinical treatments.

Since the expertise of those developing LBD tools and those using them is often different, there can be a disconnect between: 1) how developers think their tools will be used; 2) how they are actually used and perceived; and 3) the actual needs of researcher using the tool (Bekhuis, 2006). There were a number of surprises related to how our LBD tools were used and the insights we gained which we summarize here.

As mentioned in methods section, keeping terms that directly co-occur with the start term (A) term in the LBD output increases confidence in the system. In open-discovery scenarios, it is common to remove terms that directly co-occur with the start term from the LBD output. That is because LBD is looking specifically for indirect (A to C) connections and assumes that all direct (A to B) connections are already known and therefore clutter the system output with non-novel connections. We found, however that keeping direct connections in the system output increased user confidence in our system. Investigators may be unfamiliar with LBD techniques and may be skeptical of data-driven science such as LBD. Including direct connections in the LBD output provides confidence in the system by showing that it is finding meaningful information.

Removing hypotheses that are too general may not be very important. Our system represents a hypothesis as a target term and its final output was a list of target terms. Previous work (Pratt and Yetisgen-Yildiz, 2003; Hu et al., 2006) has attempted to remove terms that are too general or too obvious from this list of target terms. That is because these general terms provide no new information, and like directly co-occurring terms, they clutter the system output. We found that upon manual review of the LBD output the user quickly skimmed past terms that provided no new knowledge. This included already known and too general terms. Therefore, the inclusion of these terms in our LBD output had minimal impact on actual investigation time. Of course, a more concise list without these uninteresting terms is preferred and perhaps essential for more automated methods. We believe, however that with appropriate term ranking methods, most of these too general should rank lowly and can be removed by a threshold rather than by explicitly developing methods to explicitly remove them.

The linking terms are critical to an LBD hypothesis, and how LBD output is displayed is critical to a system’s effectiveness. Our LBD system links cardiac arrest [our starting (A) term] to diseases [our target (C) terms] via their interactions with metabolites [our linking (B) terms]. In our first iteration of the LBD system, our system output just a list diseases which we handed to the investigator. Almost immediately, the investigator asked us which metabolites linked cardiac arrest to that disease. They were interested in the whole ABC connection rather than simply a list of implicitly connected diseases. A list of implicit connections was not enough information fully explain the hypothesis. This prompted use to modify our system to output both the disease and the linking metabolites (as described in the Methods section). With that full picture of the ABC connection, the investigator quickly became excited and zeroed in on what became our discovery of LCAT as a druggable target for cardiac arrest.

Use cases are more complex than just open and closed discovery. Open discovery assumes only the start term is known, however in the process described in this paper, both the start and linking terms were already identified by the investigator. This led to the unique scenario where the A and B terms were fixed for our LBD system. Closed discovery typically requires an exact start and target term be specified, however we found that having such a well defined hypothesis is not always the case. Instead, users often have an idea of what they are looking for, but it may not be well defined. In other words, an investigator may have a vague hypothesis, such as a connection between two groupings of diseases, but the precise start and target term may not yet be known. In these cases, open discovery can be used as an exploratory tool to refine their hypothesis. By using LBD and scanning the output they can use their domain knowledge to identify patterns. These patterns can give further insight, and a process of iterative search and discovery may be used to form a more precise hypothesis.

5. LBD is useful to validate experimental findings, and can serve as a kind of automatic literature review. LBD can lead to a discovery where new associations are found in the top ranked terms. However, even the top ranked outputs which are already known to science are exciting. These support repeatable science in which the LBD results provide an unbiased validation of the findings. It allows biomedical researchers to validate their findings via a broad survey of literature rather than cherry picking a few papers which support their work, thereby supporting reproducibility in science (Smalheiser, 2017).

### 5.1 Limitations

Despite the success of our investigation, our study and LBD model did have limitations which we discuss here. These limitations pave the way for future work in developing more effective and generalizable LBD systems for metabolomic knowledge discovery.

Our LBD model is a concept based LBD model. This model relies on semantic processing to map the data to concepts in a knowledge source. This poses two disadvantages: the first is the accuracy of the semantic processing system; and the second the assumption that the knowledge source is complete. For example, in our study three metabolites highly associated with cardiac arrest did not have associated concepts in the UMLS knowledge source. This can be prohibitive for the broader adoption of LBD in other domains.

Our model utilized co-occurrence information to identify related concepts. There is disagreement as to whether co-occurrence based methods are too noisy. More complex methods such as relation extraction (Kilicoglu et al., 2020) models exist, however there is a trade-off between precision and recall with these models. Co-occurrence-based models inherently have a high recall since all co-occurrences are considered a relation, but this high recall comes at the expense of precision since many co-occurrences do not in actuality constitute a relationship. Semantic models which extract relationships from raw text pose the opposite problem. They may miss certain key relationships (lower recall), but the relationships they do extract are more likely to be true relationships. This kind of uncertainty with what constitutes a relationship, and the data that is processed is a key challenge of LBD. General purpose relation extraction algorithms often have reduced precision and recall of relation extraction algorithms for specific applications and domains. This is particularly true for the biomedical domain. Development of high recall, high precision relation extraction algorithms specifically for metabolomics would help alleviate this limitation.

Our model used fairly simple LBD techniques and focused on application driven development. As research interest in LBD has grown, more sophisticated LBD techniques have been developed. However, a major obstacle to development of these techniques, and of LBD in general has been the difficulty in objectively evaluating their performance. Many evaluation methods for LBD have been proposed, but none are perfect. Ultimately, it is impossible to predict all future discoveries, and estimating them from a dataset is fraught with questions and assumptions on what constitutes a discovery and what constitutes knowledge that is already known. See Kostoff (2002) for a discussion describing these challenges. While statistical evaluation of LBD techniques on synthetic datasets is critical for the development of new theoretical models and components of LBD systems, it is equally important to adapt these models into application. The theoretical models make assumptions about data and how it is used in practice that may not always apply to their application. Since we focused on application-driven development of an LBD system. We showed its effectiveness by its ability to generate new knowledge that is empirically evaluated. Our overall process combined previously evaluated LBD components (ABC-co-occurrence model and linking term count) adapted and integrated to a new domain. Therefore, it is not the components themselves that are novel, but rather how they are applied specifically to the task of metabolomic knowledge discovery that is novel. Therefore, we forewent any statistical evaluation such as time-slicing or link-prediction of these individual components.

Our model uses linking term count (LTC) as a target term ranking measure. We chose to use LTC because it has shown good performance in the past (Yetisgen-Yildiz and Pratt, 2009; Henry and McInnes, 2019). However, it is based solely on the count of unique linking terms shared between the start and target terms. LTC does not take the frequency of co-occurrence between A and B or B and C terms into account. In future work, we plan to experiment with using other ranking measures which may perform better for hypothesis ranking. In particular, we are interested in using indirect association measures (Henry and McInnes, 2019) which take the association between A and B and B and C terms into account when calculating target term ranks.

Our model follows the traditional ABC co-occurrence based paradigm. There has been a trend in LBD to move beyond this paradigm of term linking; for example, vector-based models avoid the ABC linking step altogether. However, due to our experimental set up in which we wanted to discover relationships between cardiac arrest and other diseases specifically through the interactions with a pre-determined set of metabolites, it is unclear how to adapt these kinds of models to this framework. While it is possible to rank all diseases (the target terms) with respect to the start term (cardiac arrest) without performing any term linking (i.e. just use all diseases as the set of target terms, then rank), the ranking method employed must take into account the interactions through our linking terms of interest. Perhaps new ranking measures could be developed to improve performance, but it is unclear whether there are benefits of going beyond ABC-type links, particularly when using co-occurrence based models. The further you move from the start term, the more noise is introduced. In our case, it is perhaps best to use ABC-type methods to generate a candidate set for manual review and put more effort into developing more advanced methods to aid in this review.

The study presented in this paper is limited to a single use case in which we used LBD to gain insights into the metabolomic processes of cardiac arrest. However, we believe that the core idea - that diseases can be better understood by gaining insights into the metabolomic processes shared between seemingly disparate diseases can be generalized to new studies. In future work, we plan to adapt the technique outlined in this paper to other diseases. Using UPLC-HRMS/MS we can identify metabolites associated with other diseases and apply a similar LBD process. Furthermore, having shown the success of this fairly simple LBD process we also hope to explore alternative LBD methods to increase the effectiveness of our system.

Although some may view the goal of LBD as a fully-automated discovery generation systems, we believe that domain expert or investigator plays a critical role in the discovery process. This outlook is similar to user-centric LBD paradigms (Cohen et al., 2010; Workman et al., 2016). We seek to develop tools that are an aid to discovery and that place the investigator in a central role in the LBD process. In this paper, we described a system that sparked the initial insight into the investigation, and while some effort was put forward to present the results in meaningful manner, our review of the generated hypotheses was almost completely manual. LBD has been posed as a two step process (Weeber et al., 2000) open-node search (open discovery) to discover new hypotheses, and a closed-node search (closed discovery) to explain these discoveries. Integration of existing visualization techniques for open discovery (Cairelli et al., 2013; Workman et al., 2016), including some of our own previous work (Henry et al., 2019), and the development and integration of closed discovery tools including visualization (Cameron et al., 2015) could benefit the process. Allowing researchers to quickly review the target terms and generate new hypotheses to explain the connections.

Lastly, while it is possible that this spark of insight may have been achieved using methods other than LBD. The goals of traditional information retrieval and LBD vastly differ. The goal of traditional information retrieval techniques focus on returning results that are most relevant based on current knowledge. LBD instead returns results which are most relevant to potential further investigation, or most relevant to potential future knowledge. While information retrieval techniques may have returned fish eye disease somewhere in their results, we believe it is very unlikely that it would be ranked highest, as it was with LBD-based techniques.

### 5.2 Conclusion

In summary, the findings from our study highlight the great potential for new knowledge discovery by directly coupling the output of metabolomic and lipidomic data for investigated diseases with the entirety of existing and up-to-date scientific literature via LBD. This work showed the efficacy of applying LBD to metabolomic/lipidomic studies. We applied fairly simple methods to make a discovery which we later empirically verified. In the future, we plan to use the discovery itself as a template for creating new LBD methods specific to the metabolomic domain. This includes more sophisticated term generation, filtering, ranking, and results display methods. Furthermore, we plan to continue to explore integrating UPLC-HRMS/MS with LBD with the specific aim of rapid analysis and verification of these metabolomic hypotheses.

## Data Availability

The datasets presented in this study can be found in online repositories. The names of the repository/repositories and accession number(s) can be found below: https://github.com/Scientific-LBD/metabolite-LBD-cardiac-arrest.

## Footnotes

1
https://metamap.nlm.nih.gov/SemanticTypesAndGroups.shtml

2
https://mbr.nlm.nih.gov/

3
https://ii.nlm.nih.gov/MMBaseline/index.shtml

4 Code is available: https://github.com/Scientific-LBD/metabolite-LBD-cardiac-arrest

5
https://github.com/Scientific-LBD/metabolite-LBD-cardiac-arrest
